# INF-γ Enhances Nox2 Activity by Upregulating phox Proteins When Applied to Differentiating PLB-985 Cells but Does Not Induce Nox2 Activity by Itself

**DOI:** 10.1371/journal.pone.0136766

**Published:** 2015-08-28

**Authors:** Michael A. Ellison, Gail Thurman, Christy M. Gearheart, Ryan H. Seewald, Christopher C. Porter, Daniel R. Ambruso

**Affiliations:** 1 Department of Pediatrics, University of Colorado School of Medicine, Aurora, CO, United States of America; 2 Department of Pediatrics, University of Colorado Denver, The Anschutz Medical Campus, Aurora, Colorado, United States of America; 3 Department of Pathology, University of Colorado Denver, The Anschutz Medical Campus, Aurora, Colorado, United States of America; 4 The Center for Cancer and Blood Disorders, Transfusion Services, Children's Hospital Colorado, Aurora, Colorado, United States of America; 5 Hematology/Oncology and Bone Marrow Transplantation Laboratories, Aurora, Colorado, United States of America; Rutgers - New Jersey Medical School, UNITED STATES

## Abstract

**Background:**

The cytokine and drug interferon-γ enhances superoxide anion production by the antimicrobicidal Nox2 enzyme of neutrophils. Because mature neutrophils have a short lifespan, we hypothesized that the effects of interferon-γ on these cells might be mediated by its prolonged exposure to differentiating neutrophil precursors in the bone marrow rather than its brief exposure to mature circulating neutrophils.

**Effects of INF-γ on Nox2 Activity:**

To address this possibility we exposed the myeloid PLB-985 cell line to interferon-γ for 3 days in the presence of dimethyl sulfoxide which induces terminal differentiation of these cells. Interferon-γ was found to enhance superoxide production by Nox2 in a concentration dependent manner. In contrast, application of interferon-γ alone for 3 days failed to induce detectible Nox2 activity. Additionally, application of interferon-γ for 3 hours to pre-differentiated PLB-985 cells, which models studies using isolated neutrophils, was much less effective at enhancing superoxide anion production.

**Effects of INF-γ on phox Protein Levels:**

Addition of interferon-γ during differentiation was found to upregulate the Nox2 proteins gp91phox and p47phox in concert with elevated transcription of their genes. The p22phox protein was upregulated in the absence of increased transcription presumably reflecting stabilization resulting from binding to the elevated gp91phox. Thus, increased levels of gp91phox, p47phox and p22phox likely account for the interferon-γ mediated enhancement of dimethyl sulfoxide-induced Nox2 activity. In contrast, although interferon-γ alone also increased various phox proteins and their mRNAs, the pattern was very different to that seen with interferon-γ plus dimethyl sulfoxide. In particular, p47phox was not induced thus explaining the inability of interferon -γ alone to enhance Nox2 activity. Short application of interferon-γ to already differentiated cells failed to increase any phox proteins.

**Conclusions:**

Our findings indicate that interferon-γ has complex effects on phox protein expression and that these are different in cells undergoing terminal differentiation. Understanding these changes may indicate additional therapeutic uses for this cytokine in human disorders.

## Introduction

Named for their potent ability to interfere with and protect against viral infections, interferons (IFNs) are cytokines that have many regulatory effects on the immune system [1]. Of the members of the two classes of these compounds, IFN-γ, the only member of the Type II IFN family, has the most diverse and powerful immune activities.

To date, studies of IFN-γ have mostly evaluated its interactions with cells of adaptive immunity, including macrophages and lymphocytes. Effects on innate immunity, particularly neutrophils/polymorphonuclear leucocytes (PMNs) and monocytes, are less well defined. The data that does exist for PMNs suggest that IFN-γ may be involved in modulating their signal transduction, gene expression, phagocytosis, motility and apoptosis as well as the generation of microbicidal superoxide anion (O_2_^-^) by the phagocyte oxidase/Nox2 enzyme [1]. Not all of these functions are enhanced by IFN-γ, for example it may reduce cell motility. The clinical use of this cytokine has been driven in part by these findings. For example IFN-γ is used to treat Chronic Granulomatous Disease (CGD), an inherited disorder of the bactericidal activity of phagocytic cells in which deficiency of a protein subunit (phox protein) of Nox2 leads to an inactive enzyme with a reduced or abolished ability to produce superoxide anion [2]. The primary motivation for investigating the clinical effects of IFN-γ in CGD was its experimentally observed enhancement of O_2_^-^ production by Nox2 [2] suggesting that patients with reduced expression of a phox protein might benefit from IFN-γ administration. Most existing data in this area is based on studies using brief exposures of IFN-γ to short lived mature PMNs from peripheral blood [1]. However, since PMNs undergo a prolonged maturation process in the bone marrow before release into circulation, it seems that it is important to define the phenotype of PMNs that have been matured in the presence of IFN-γ. This phenotype, rather than just changes mediated by brief IFN-γ application to mature PMNs may be critical to understanding the physiologic effects of this cytokine and to expanding its use into the treatment of a broader range of human diseases.

To enhance our understanding of the role of IFN-γ in the development and functional integrity of the PMN, we made use of PLB-985 cells in an *in vitro* culture system. This myeloid cell line can be matured toward a PMN like state using various agents [3,4]. This results in the development of an active Nox2 enzyme including all the essential protein subunits and PLB-985 cells have thus been used as a model to explore Nox2 development and function.

In this study we looked specifically at changes in Nox2 activity and the levels of its constituent proteins that occur when IFN-γis applied *during* PLB-985 cell maturation. The Nox2 proteins examined were the membrane associated p22phox and gp91phox (which form the cytochrome b558 heterodimer) and the cytoplasmic Nox2 subunits, p40phox, p47phox and p67phox which bind to cytochrome b558 upon Nox2 activation. When Nox2 is activated the resulting holoenzyme transfers electrons from cytoplasmic NADPH to phagolysosomal or extracellular O_2_ thus forming O_2_^-^. This initiates the production of a cascade of reactive oxygen species (ROS) which are, in part, responsible for the microbicidal activity of myeloid cells [2].

Using PLB-985 cells we established a cell culture model which approximates *in vivo* myeloid cell development and determined the effects of IFN-γ on Nox2 activity and phox protein expression in this system. We also examined Nox2 activity and phox protein expression in PLB-985 cells following application of IFN-γ in the absence of differentiation. Additionally, to model previous studies using isolated neutrophils, we explored the effect on Nox2 activity and phox protein expression of short term application of IFN-γ to already differentiated PLB-985 cells.

## Methods

### Cell Culture, Cell Differentiation and Treatment with IFN-γ

PLB-985 cells from the German Collection of Microorganisms and Cell Cultures (DSMZ) were grown at 37°C in suspension in RPMI 1640 medium containing 2mM L-glutamine (Gibco), 10% bovine serum (Atlanta Biologicals) and 100 units/mL penicillin plus 100 mg/mL streptomycin (Sigma Life Science). The cultures were maintained in a humidified, 5% CO_2_ atmosphere. The cells were split to 0.2X10^6^/ml every 3 to 4 days. The cells would double in slightly longer than 24 hours and reach about 3.0X10^6^/ml before reductions in growth rate were seen.

PLB-985 cells (1–1.5X10^6^/ml) in log phase growth were pelleted (250XG, 5min) and re-suspended to 0.4X10^6^/ml in the standard media or the standard media plus 1.3% (vol:vol) DMSO (Sigma-Aldrich), in the presence or absence of various concentrations of IFN-γ (Horizon Pharma Ireland Ltd). These cells were used in experiments after 72 hours. In some experiments, cells differentiated for 72 hours in 1.3% DMSO were pelleted and resuspended in fresh media and were then exposed to various concentrations of IFN-γ, for a further 3 hours.

Light microscopy was used to determine cell number and, by trypan blue (Gibco) exclusion, cell viability. After 72 hours incubation in media, media with 30ng/ml IFN-γ, media with 1.3% DMSO or media with 1.3% DMSO plus 30ng/ml IFN-γ non-viable cells were 5+/-2%, 17+/-9%, 16+/-6% and 15+/-6% (mean +/- SD, n at least 6) of the total respectively. In the pre-differentiated cells exposed for 3 hours to 30ng/ml IFN-γ or to media alone, trypan blue permeable cells were 18+/-8% and 16+/-7% (mean +/- SD, n = 4) of the total respectively. Thus, the conditions used in this study did not induce large amounts of cell death which could have affected the measured cell properties.

PLB-985 cells are a subclone of the HL-60 line. Despite being described as isolated years apart from different patients, HL-60 [5] and PLB-985 [3] cells are now known to be from the same individual [6], indicating that the original “isolation” of PLB-985 cells probably reflects expansion of an HL-60 cell contamination. Nevertheless, according to the original descriptions of PLB-985 and HL-60 cells, the two lines have very different granule morphology and distinct susceptibilities to cytological stains which suggests that PLB-985 cells are less matured along the myeloid lineage than HL-60 cells [3]. This phenotype may have been acquired over time in lab culture but is nevertheless consistent with PLB-985 cells being a model for a more primitive precursor along the myeloid lineage. In our hands a more primitive PLB-985 phenotype relative to HL-60 cells (DSMZ) was confirmed by the observation that PLB-985 cells have less myeloperoxidase. Furthermore, about 80% of Wright-Giemsa stained PLB-985 cells have a myeloblast morphology and about 20% have a promyelocyte or myelocyte morphology. In contrast, only about 30% of identically stained HL-60 cells have a myeloblast morphology and about 65% have a promyelocyte or myelocyte morphology; this finding is similar to what others have described [7]. Furthermore we also find that the two cell lines are phenotypically different with respect to their responses to IFN-γ. As described below, this study reveals that PLB-985 cells treated with IFN-γ alone do not acquire Nox2 activity in contrast to what has been described for HL-60 cells [8,9]. We confirmed this difference between the two cell lines finding that HL-60 cells treated with IFN-γ (as described below for PLB-985 cells) produce superoxide in response to the Nox2 agonists phorbol 12-myristate 13-acetate (PMA). Superoxide anion production stimulated by this agonist in HL60 cells, and measured as described below for PLB-985 cells, was 9.7+/-2.4 nmol (average +/-SEM, n = 3).

### Measurement of Nox2 Activity

O_2_^-^ production by PLB-985 cells was measured spectrophotometrically in a plate reader by following SOD inhibitable reduction of cytochrome C as described previously for PMNs [10,11].

### Western Blotting

PLB-985 cells were pelleted at 250XG for 5min, were resuspended in 1ml of ice cold KRP-d (12.5 mM Na_2_HPO_4_, 3 mM NaH_2_ PO_4_, 4.8 mM KCl, 120 mM NaCl, 1.3 mM CaCl_2_, 1.2 mM MgSO_4_, 0.2% dextrose, pH 7.3–7.4) and were re-centrifuged with the resulting pellets being stored stored at -70°C until use. After thawing, cells were resuspended in 1ml of lysis buffer (150mM NaCl, 50mM Tris-HCl, 50mM NaF, 5mM EDTA, 0.5% (weight:volume) sodium deoxycholate, 0.1% (weight:volume) sodium dodecyl sulfate (SDS), 1% (volume:volume), Triton X-100, pH7.6) containing a protease inhibitor mix obtained by dissolving EDTA free-cOmplete Ultra Tablets (Roche) according to the manufacturer’s instructions. Cells were the disrupted by sonication for 30 seconds at 30% power in a model W-220F cell disruptor (Heat Systems-Ultrasonics Inc.).

Proteins from cell lystes (50μg for p22phox, p40phox and gp91phox, 25μg for p67phox and 10μg for p47phox) were resolved by SDS-PAGE. Proteins were transferred (400 mA, 1 hour) to activated polyvinylidene flouride membranes (for gp91phox) or nitrocellulose membranes (for all other proteins) in buffer containing 20% (volume:volume) methanol, 0.025 M glycine, and 0.0015% (volume:volume) ethanolamine.

For detection of proteins, membranes were exposed to antibodies diluted in 10% (weight:volume) non-fat dry milk (NFDM) in TBS-T (20mM Tris, 137mM NaCl, 0.1% (volume:volume) Tween-20, pH 7.6). The following primary antibodies were applied overnight at 4°C and a 1:1000 dilution; mouse anti-gp91 (Santa Cruz Biotechnology sc-130543); rabbit anti-p67 (Millipore 07–002); goat anti-p47 (a gift from Dr. TL Leto that has been described previously, [12]); rabbit anti-p40 (US Biological P1001-20C) and rabbit anti-p22 (Santa Cruz Biotechnology sc-20781). The secondary antibodies were peroxidase linked anti-rabbit IgG or anti-mouse IgG (both from GE Healthcare) or peroxidase linked anti-goat IgG (Rockland Immunochemicals Inc.) as appropriate. To establish a loading and transfer efficiency control, all the membranes were re-probed with a mouse anti-GAPDH primary antibody (Santa Cruz Biotechnology sc-47724) for 1 hour at room temperature and a peroxidase linked anti-mouse, IgG secondary antibody (1 hour, room temperature). Immune complexes were then detected with an enhanced chemiluminescence (ECL) detection system (GE Healthcare) according to the manufacturer’s instructions. All chemicals for western blotting were from Sigma-Aldrich, Acros or Fisher.

Quantitation of proteins detected by western blotting was done by densitometry using ImageJ software (http://imagej.nih.gov/ij/).

### Plasma Membrane Preparation

Plasma membrane was prepared as described earlier [13]. Briefly, PLB-985 cells were treated with IFN-γ (30ng/ml) and/or DMSO, or were left untreated, as described above. The cells were then resuspended to 1X10^8^/ml in relaxation buffer (10mM Pipes, 3mM NaCl, 100mM KCL, 3.5mM MgCl2, 1.2mM EGTA, pH7.4) containing the same protease inhibitor cocktail described earlier. Cells were sonicated (3X15 seconds at 30% power with rests on ice) using the cell disruptor described earlier. Unbroken cells were pelleted (250XG, 5 minutes) and the supernatant (about 1ml) was layered on top of a discontinuous sucrose gradient in relaxation buffer (2ml (15%), 9.5ml (40%), in a 12.5 ml ultracentrifuge tube). After centrifugation (100,000XG, 1 hour) membrane was removed from above the 40% layer and frozen at -70^°^C until used for western blotting; membrane fractions containing 4μg of total protein were loaded per well to detect membrane associated Nox2 proteins.

### Microarray Analysis of phox Gene mRNA

PLB-985 cells were treated as described under “Cell differentiation and treatment with IFN-γ” (30ng/ml IFN-γ was used). Aliquots containing one million cells were pelleted and stored at -70°C until use. Total RNA was extracted using the RNeasy Mini Kit (Qiagen).

All further microarray work was done at the University of Colorado Genomic and Microarray Core Facility. Preparation of labelled cDNA, hybridization to HuGene2.0ST microarrays (Affymetrix), microarray washing and staining (using a GeneChip Fluidics Station 450) and microarray scanning (using a GeneChip Scanner 3000) were done as recommended by the manufacturer in the corresponding protocols (GeneChip WT PLUS Reagent Kit, AffymetrixGeneChip Fluidics Station 450 User's Guide, the GeneChip Expression Wash, Stain, and Scan User Manual for Cartridge Arrays (PN 702731), and the Affymetrix GeneChip Command Console User Manual).

Data collected from microarray analysis were preprocessed using the Affymetrix Expression Console (Build 1.3.1.187) to be RMA-normalized then log2 transformed to minimize technical variation among samples across multiple arrays [14]. Hierarchical clustering was performed using the complete linkage method of Euclidean distances of log2-transformed data within the Affymetrix Transcriptome Analysis Console (Version 2.0.0.9). Relative expression levels were then compared. One-way unpaired ANOVA analysis of 48,226 genes found 4,121 genes to be differentially expressed, defined as having at least a two-fold linear over- or under-expression with an associated p-value less than 0.05.

For this study mRNA from 4 independent experiments was analyzed.

## Results

### Nox2 Activity of PLB-985 Cells after Exposure to DMSO, IFN-γ, or DMSO plus IFN-γ

**Fig 1:**
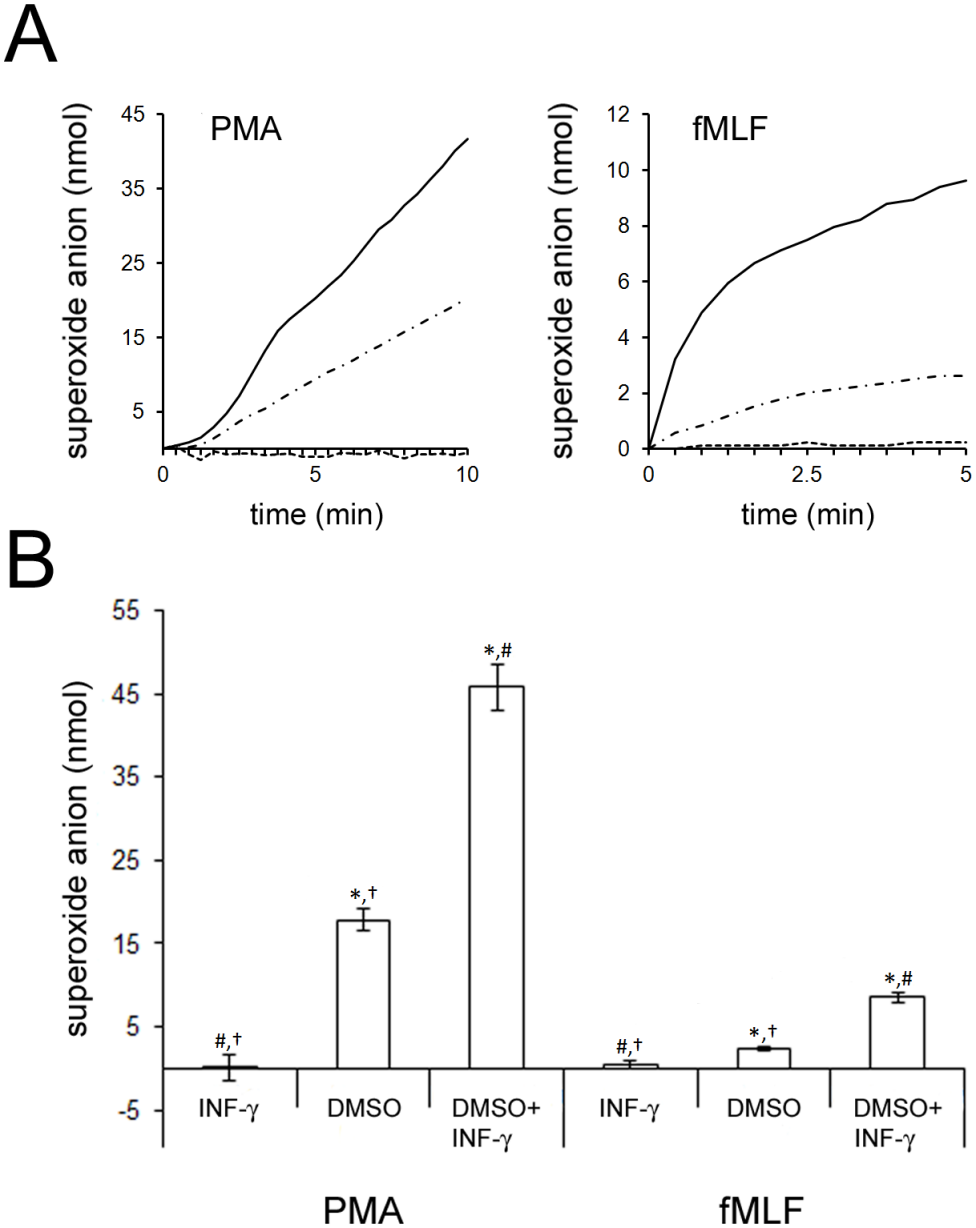
Effect of IFN-γ on PLB-985 Cell Nox2 Activity in the Presence and Absence of Differentiation. A; PLB-985 cells were pretreated for 72 hours with 1.3% DMSO, 30ng/ml IFN-γ or 1.3% DMSO plus 30ng/ml IFN-γ. The cells were stimulated with the Nox2-agonists PMA or fMLF, as indicated, and O_2_^-^ generation was followed over time (DMSO plus IFN-γ, black lines; DMSO, dots and dashes; IFN-γ, dashes). Representative traces are shown. When SOD was included no superoxide generation was detected for any combination of pretreatment or agonist (not shown). B; The amount of O_2_^-^ formed in 10 minutes after addition of PMA, or 5 minutes after addition of fMLF, was recorded during multiple experiments like those shown in A. The graph shows the average O_2_^-^ produced (error bars are +/- SEM) for at least 6 experiments carried out after at least 3 independent pretreatments with DMSO, IFN-γ or DMSO plus IFN-γ Statistical differences with in the three cell culture conditions subsequently treated with PMA or those treated with fMLF were confirmed by one way ANOVA and significance between each pair was then explored by post hoc-FDR analysis of paired t-tests. *, # and † indicate significant difference from IFN-γ, DMSO and DMSO+IFN-γ treatment respectively (p<0.05).

As shown in Fig 1A, a 72 hour exposure of PLB-985 cells to 30ng/ml IFN-γ did not result in detectible O_2_^-^ production when PLB-985 cells were stimulated with the Nox2 agonists PMA or N-formylmethionine-leucine-phenylalanine (fMLF). Maintenance of PLB-985 cells in media alone also resulted in no detectible O_2_^-^ production (data not shown). In contrast, 72 hours of DMSO treatment resulted in a sustained respiratory burst in response to PMA and an initially faster but then plateauing production of O_2_^-^ in response to fMLF. This is consistent with the well-described ability of DMSO to cause terminal differentiation of PLB-985 cells toward a PMN-like state that includes functional Nox2 [4]. Strikingly, a 72 hour co-incubation of IFN-γ and DMSO enhanced O_2_^-^ generation relative to DMSO application alone.

Fig 1B summarizes the aggregate results from 6 experiments like those in Fig 1A. The increase in Nox2 generated O_2_^-^ in response to fMLF and PMA was significant for DMSO treated cells relative to IFN-γ treated cells. Furthermore, the increase in O_2_^-^ production was significant for both Nox2 agonists when comparing DMSO plus IFN-γ treatment to IFN-γ treatment and to DMSO treatment.

Additional studies like those summarized in Fig 1 were done using 96 hour instead of 72 hour cell incubations and the relative levels of Nox2 activity showed the same pattern (data not shown).

Together the results indicate that 30ng/ml IFN-γ does not by itself induce Nox2 activity in PLB-985 cells; however, it can enhance Nox2 activity resulting from DMSO mediated differentiation of these cells.

In further studies we added DMSO to PLB-985 cells for 72 hours as described in Methods but then added IFN-γ, at 30ng/ml, after 24 and 48 hours of DMSO treatment. Nox2 activity stimulated with fMLF and PMA was then measured. For both agonists, the 1 and 2 day co-applications of DMSO and IFN-γ resulted in superoxide anion production that was no different (p>0.05, two tailed t-test, n at least 3) from that caused by the 3 day co-applications described above. Thus enhancement of Nox2 activity by IFN-γ during DMSO-mediated PLB-985 differentiation is rapid and occurs in less than 24 hours.

We also explored the effect of pre-application of IFN-γ. PLB-985 cells were treated with IFN-γ for 72hr as described earlier, the same cells were then treated for 72 hours with IFN-γ plus DMSO as described in Methods. No difference (p>0.05, two tailed t-test, n = 4) was seen in Nox2 activity (fMLF or PMA stimulated) in these cells relative to cells that were only subjected to 72 hours of co-application of IFN-γ plus DMSO. This indicates that pretreatment with IFN-γ alone does not modulate the observed enhancement of Nox2 activity caused by the presence of IFN-γ during DMSO mediated differentiation.

### IFN-γ Applied During, or After, Differentiation of PLB-985 Cells has Concentration Dependent Effects on Nox2 Activity

**Fig 2:**
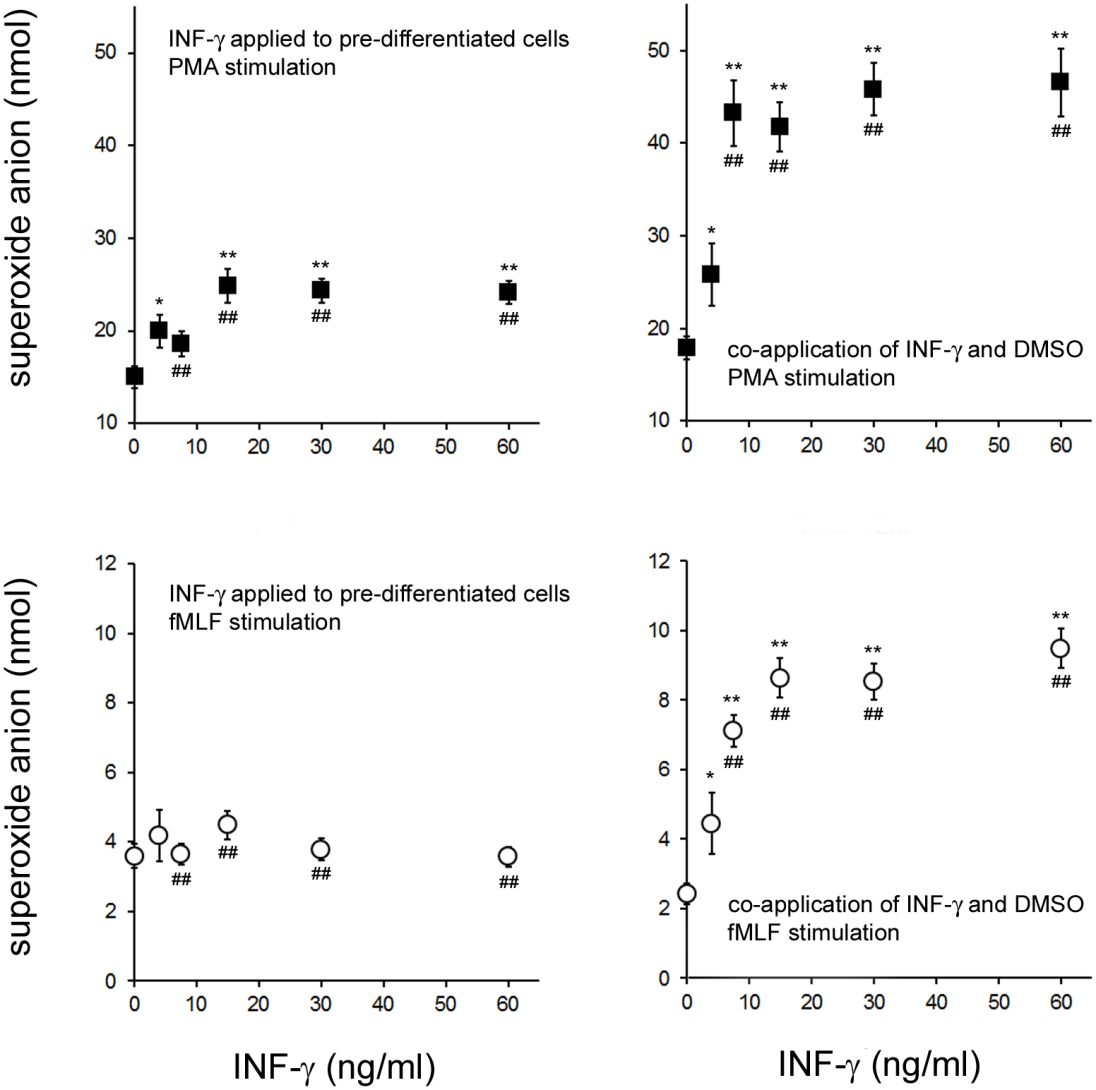
Stimulation of PLB-985 Nox2 Activity when IFN-γ was Applied After or During Cell Differentiation. In the two graphs on the left, PLB-985 cells were treated for 72 hours with 1.3% DMSO (pre-differentiated) and were then treated for 3 hours with the indicated concentrations of IFN-γ. The cells were then stimulated with PMA (top graph) or fMLF (bottom graph) and the amount of O_2_^-^ generated in 10 minutes (for PMA) or 5 minutes (for fMLF) was measured. In the two graphs on the right, PLB-985 cells were cultured for 72 hours in the presence of 1.3% DMSO and simultaneously treated with the indicated concentrations of IFN-γ(co-application). The cells were then stimulated with the Nox2 agonists PMA (top graph) or fMLF (bottom graph) and the amount of O_2_^-^ generated after 10 minutes (for PMA) or 5 minutes (for fMLF) was measured. The error bars are +/- SEM and n = at least 12 measurements from at least 2 independently treated cultures of PLB-985 cells. For a given graph, * indicates a significant difference (p<0.05; two tailed t-test) and ** indicates a very significant difference (p<0.005; two tailed t-test) between O_2_^-^ produced at a given IFN-γ concentration and O_2_^-^ formed in the corresponding 0ng/ml-IFN-γ condition. For a given agonist (PMA or fMLF), ## indicates a very significant difference (p<0.005; two tailed t-test) between the increase in O_2_^-^ formed due to a given IFN-γ concentration and the equivalent increase in the alternative IFN-γ application protocol (co-application of IFN-γ and DMSO versus IFN-γ application to pre-differentiated cells).

When IFN-γ was applied for 3 hours to PLB-985 cells that had been pre-differentiated by 72 hours in DMSO, PMA stimulated O_2_^-^ production increased with IFN-γ concentration eventually reaching a plateau (Fig 2, top left panel). Maturation of the PLB-985 cells (72 hours in DMSO) in the presence of IFN-γ also resulted in an increase in PMA mediated O_2_^-^ generation (Fig 2, top right panel) which was dependent on IFN-γ concentration. In this case however, the plateau in O_2_^-^ production was at a higher level than when IFN-γ was applied for 3 hours to pre-differentiated PLB-985 cells.

In contrast to what was seen with PMA stimulation, IFN-γ was not associated with a significant increase in fMLF stimulated O_2_^-^ generation when applied for 3 hours to pre-differentiated PLB-985 cells (Fig 2, bottom left panel). However, co-application of IFN-γ with DMSO (Fig 2, bottom right) did result in an increase in O_2_^-^ production that was dependent on IFN-γ concentration.

### IFN-γ Alters Whole-Cell phox Protein Expression When Present During PLB-985 Differentiation

**Fig 3:**
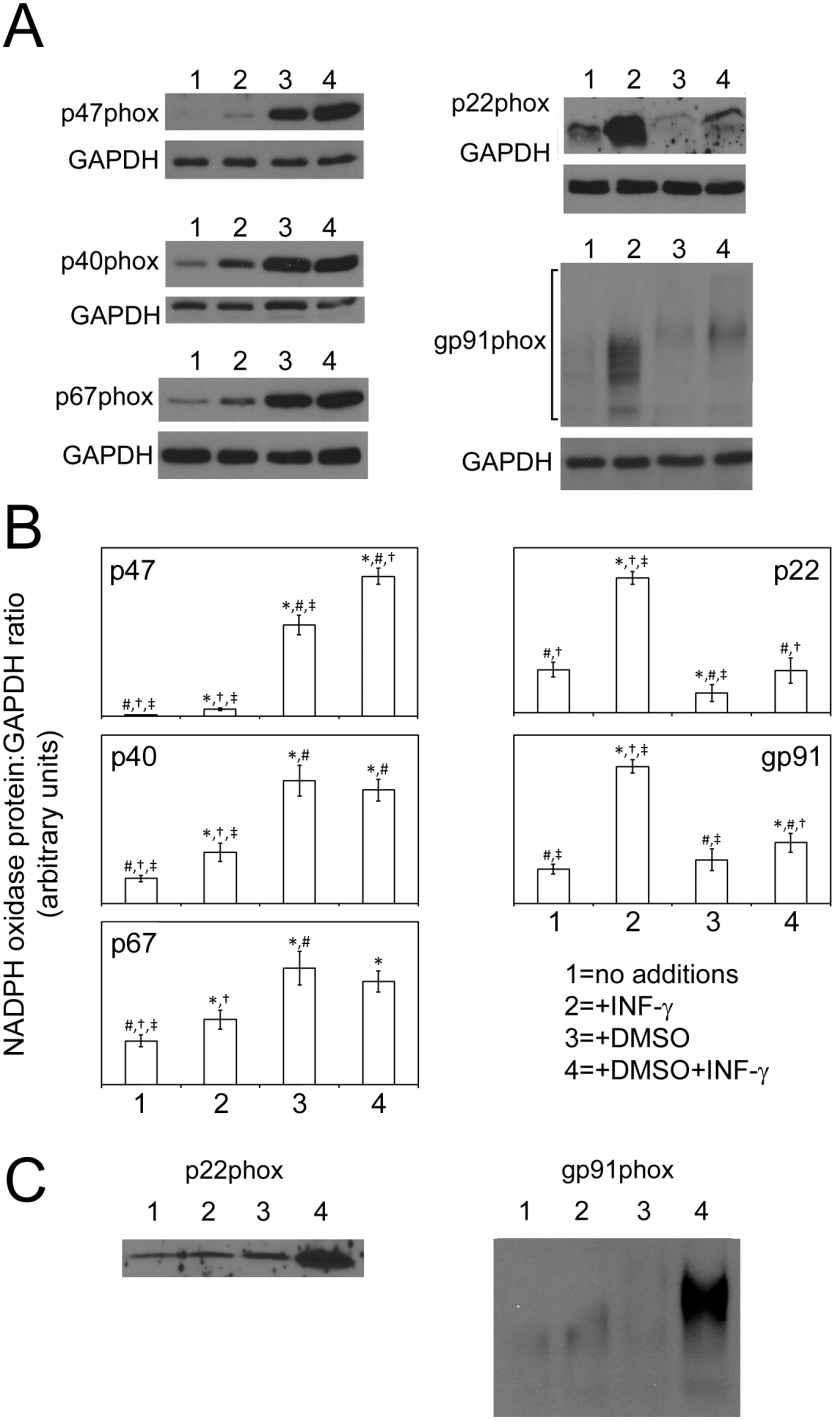
PLB-985 phox Proteins in Cells Exposed to IFN-γ in the Presence or Absence of Differentiation. A; The western blots are from representative experiments and show the indicated Nox2 proteins in PLB-985 cells that were cultured for 72 hours in the presence of media with, no additions (lane 1), 30ng/ml IFN-γ (lane 2), 1.3% DMSO (lane 3) or 1.3% DMSO plus 30ng/ml IFN-γ (lane 4). Blots were reprobed for GAPDH to establish controls for gel loading and transfer efficiency. For gp91phox the protein was detected over a broad range of molecular weights (indicated by the square bracket) due to glycosylation. B; The graphs show the average density of phox proteins measured in 4 experiments like the representative examples in A; the protein densities are expressed as a ratio to the corresponding GAPDH density. For densitometry of glycosylated and thus heterogeneously sized gp91phox, the lanes were scanned to the extent indicated by the square bracket in the representative gp91phox blot. The error bars are +/- SEM. For each protein, statistical differences with in the different cell culture conditions were confirmed by one way ANOVA and significance between each pair was then explored by post hoc-FDR analysis of paired t-tests. *, #, † and ‡ indicate significant difference from untreated cells (“no additions”), IFN-γ treatment, DMSO treatment and DMSO+IFN-γ treatment respectively (p<0.05). C; The western blots show the indicated Nox2 proteins in subcellular membrane preparations from PLB-985 cells that were cultured as described in panel A. The blots are representative of 2 independent experiments.

Western blotting of cell lysates was used to determine the effect of prolonged (72 hour) applications of IFN-γ (30ng/ml), DMSO and DMSO plus IFN-γ on whole-cell Nox2 proteins levels. As shown in Fig 3, three patterns of response were observed, one pattern was shared by the membrane bound Nox2 proteins gp91phox and p22phox, one was shared by p67phox and p40phox and one was unique to p47phox.

#### Pattern for gp91phox and p22phox

For both gp91phox and p22phox, 72 hour applications of IFN-γ caused a large increase in protein levels (about 4 fold for gp91phox and about 3 fold for p22phox) relative to untreated cells.

72 hour incubations with DMSO alone resulted in a minimal reduction of p22phox relative to untreated cells but there was no statistically significant change in gp91phox protein.

When IFN-γ was applied with DMSO, a reduction in the IFN-γ mediated increase of p22phox and gp91phox was seen; in this case the increases were only about 1.4 fold for gp91phox and about 2 fold for p22phox (when comparing DMSO plus IFN-γ to DMSO treated cells). Despite their relatively smaller magnitude, the increases in gp91phox and p22phox were still statistically significant.

A further effect of DMSO on gp91phox was apparent alteration of its glycosylation pattern. On the gp91phox blot in Fig 3A it can be seen that inclusion of DMSO shifts gp91phox to a larger size presumably reflecting more extensive polysaccharide structures on the protein. IFN-γ did not change the observed pattern off glycosylation in either the presence or absence of DMSO.

#### Pattern for p40phox and p67phox

A second pattern of changes in protein level was seen with the cytoplasmic Nox2 components p40phox and p67phox. IFN-γ alone caused an approximately 2 fold increase in p40phox and an approximately 1.5 fold increase in p67phox relative to untreated cells. For both proteins DMSO increased their levels by a large amounts relative to cells maintained in media alone (about 5 fold for p40phox and 3 fold for p67phox). However the presence of DMSO also abolished the ability of IFN-γ to increase the levels of these proteins; unlike when the effects of media alone and media plus IFN-γ are compared, there is no difference in p40phox or p67phox when the effects of DMSO and DMSO plus IFN-γ are compared.

#### Pattern for p47phox

A final pattern of behavior was seen with p47phox. For this protein IFN-γ caused a minimal increase in its level relative the very low amount in cells maintained in media alone. DMSO caused a large increase in p47phox levels and co-application of DMSO and IFN-γ further enhanced this increase.

### IFN-γ Increases p22phox and gp91phox Levels in PLB-985 Plasma Membrane when Applied to Differentiating Cells

In functional Nox2, electrons cross the membrane by means of a transmembrane p22phox-gp91phox heterodimer. Therefore, to confirm that the whole-cell-increases in p22phox and gp91phox described above translate into corresponding increases in membrane associated protein we treated PLB-985 cells for 72 hours with IFN-γ (30ng/ml), DMSO and DMSO plus IFN-γ, disrupted the cells and used western blotting to detect p22phox and gp91phox in isolated subcellular plasma membrane preparations. As can be seen in Fig 3C, inclusion of IFN-γ with DMSO caused large increases in membrane associated p22phox and gp91phox relative to DMSO alone, this is consistent with the pattern for these proteins in whole-cell lysates. In contrast, when comparing cells grown in the presence of IFN-γ to those grown in media alone, changes in the membrane levels of these proteins were minimal compared to their corresponding large increases in whole-cell lysates.

We also determined p47phox levels in the membrane preparations. Consistent with the fact that it is a cytosolic Nox2 protein that only becomes associated with the membrane upon Nox2 activation we found very low levels of it in all conditions; p47phox was several hundred fold less abundant in plasma membrane preparations than in whole cell lysates from equivalent numbers of cells (data not shown).

### IFN-γ Alters mRNA Levels of phox Proteins When Present During PLB-985 Differentiation

To explore whether the observed changes in phox protein level described in the previous sections were related to changes in transcription of the corresponding genes we made use of some of the data from an ongoing study to examine transcriptome wide changes in PLB-985 mRNA levels in response to IFN-γ, DMSO and DMSO plus IFN-γ. RNA samples from PLB-985 cells treated with these agents, or untreated controls, were analyzed using Human Genome 2.0 microarrays. This allowed the corresponding fold changes in the mRNA levels of Nox2 subunits to be inferred (Table 1).

**Table 1:** Pairwise Changes in phox Protein mRNA Levels when PLB-985 Cells were Maintained for 72 Hours in the Presence or Absence of DMSO and/or IFN-γ.

Protein	Fold Change	p-value
mRNA change in IFN-γ treated cells relative to untreated cells		
p22phox	1.05	0.68
gp91phox	2.48	0.000015
p47phox	1.14	0.52
p67phox	1.68	0.0033
p40phox	1.13	0.069
mRNA Change in DMSO treated cells relative to untreated cells		
p22phox	2.18	0.00048
gp91phox	3.47	5.0X10^−7^
p47phox	5.3	0.0000070
p67phox	6.89	7.3X10^−7^
p40phox	2.51	0.0000040
mRNA change in DMSO plus IFN-γ treated cells relative to untreated cells		
p22phox	2.07	0.00083
gp91phox	4.14	3.3X10^−7^
p47phox	8	0.000011
p67phox	7.33	7.2X10^−7^
p40phox	2.45	0.0000020
mRNA change in DMSO plus IFN-γ treated cells relative to DMSO treated cells		
p22phox	-1.05	0.098
gp91phox	1.19	0.010
p47phox	1.51	0.025
p67phox	1.06	0.34
p40phox	-1.02	0.86

Pairwise comparisons of the changes in phox protein mRNA levels after 72 hours of culture in various conditions. RNA measurement was done using Affymetrix Human Genome 2.0 DNA microarrays. For each phox protein, the inferred fold change in mRNA is shown for the indicated comparisons along with the corresponding statistical significance. The p values were obtained from an ANOVA test. The data is based on 4 independent experiments in which all 4 cell-treatment conditions were carried out.

#### mRNA changes in IFN-γ treated cells relative to untreated cells

As can be seen in Table 1, treatment with 30ng/ml of IFN-γ resulted in a large increase of gp91phox mRNA. This is consistent with the large increase in whole-cell gp91phox protein observed by western blot (Fig 3A and 3B). Although p22phox protein levels increased substantially with IFN-γ treatment (Fig 3A and 3B), no corresponding increase in p22phox mRNA was seen (Table 1). The increase in p22hox protein in the presence of IFN-γ could thus be due to increased protein stabilization or elevated translation of p22phox mRNA in the presence of IFN-γ. Table 1 also reveals that IFN-γ treatment caused an approximately 70% increase in p67phox mRNA which is consistent with the significant increase in p67phox protein observed upon IFN-γ application (Fig 3). In contrast, although p40phox protein increased by roughly the same amount as p67phox upon IFN-γ treatment, no corresponding increase in p40phox mRNA was observed (Table 1) indicating that IFN-γ enhances p40phox stability or stimulates its translation. There was no significant increase in p47phox mRNA (see Table 1) upon IFN-γ treatment suggesting that the very small but statistically significant increase in p47phox protein upon IFN-γ treatment (see Fig 3.) might also be due to enhanced protein stability of increased translation of the p47phox mRNA.

#### mRNA changes in DMSO treated cells relative to untreated cells

Table 1 indicates that treatment of PLB-985 cells with DMSO caused large and very statistically significant increases in the levels of all phox protein mRNAs relative to no treatment. For the cytoplasmic proteins p40phox, p47phox and p67phox this correlates well with the large increases in their protein levels (see Fig 3A and 3B). However, for gp91phox and p22phox, despite the large increases in mRNA, protein levels in whole cell lysates were down slightly (for p22phox) or unchanged (for gp91phox) relative to untreated cells (Fig 3A and 3B). This indicates that the reduction in the cytochrome b558 heterodimer caused by DMSO is either mediated post-transcriptionally, possibly because of increased protein instability, or is due to reduced translation of gp91phox and p22phox mRNAs.

#### mRNA changes in DMSO plus IFN-γ treated cells relative to untreated cells and in DMSO plus IFN-γ treated cells relative to DMSO treated cells

As shown in Table 1, PLB-985 cells treated with DMSO plus IFN-γ display large increases in the levels of all phox protein mRNAs relative to untreated cells. For gp91phox and p47phox the increases in mRNA levels were larger than the corresponding increases with DMSO alone; gp91phox mRNA was elevated about 50% relative to DMSO alone and p47phox mRNA was up almost 20%. These increases in mRNA likely account for the increases in gp91phox and p47phox protein, relative to treatment with DMSO alone, when IFN-γ is added during differentiation (see Fig 3). As shown in Fig 3, p22phox protein was also elevated in cells treated with DMSO plus IFN-γ relative to cells treated with DMSO alone. However, there was no corresponding increase in p22phox mRNA (Table 1) indicating that the increase in p22phox protein might be due to its increased protein stabilization or enhanced translation of p22phox mRNA.

### IFN-γ has Minimal Effects on Nox2 Protein and mRNA Levels When Applied Briefly to Pre-Differentiated PLB-985 Cells

Previous studies have shown that when mature PMNs from peripheral blood are treated for several hours with IFN-γ they display an enhanced respiratory burst [15,16]. In our current study a similar situation was modeled by treating pre-differentiated PLB-985 cells with IFN-γ for 3 hours prior to activation of the respiratory burst. As shown in Fig 2, this resulted in a concentration dependent increase in PMA stimulated O_2_^-^ production by the cells that was less than the corresponding increase resulting from prolonged co-application of DMSO and IFN-γ. fMLF stimulated a small but not statistically significant increase in superoxide anion production at lower IFN-γ concentrations.

**Fig 4:**
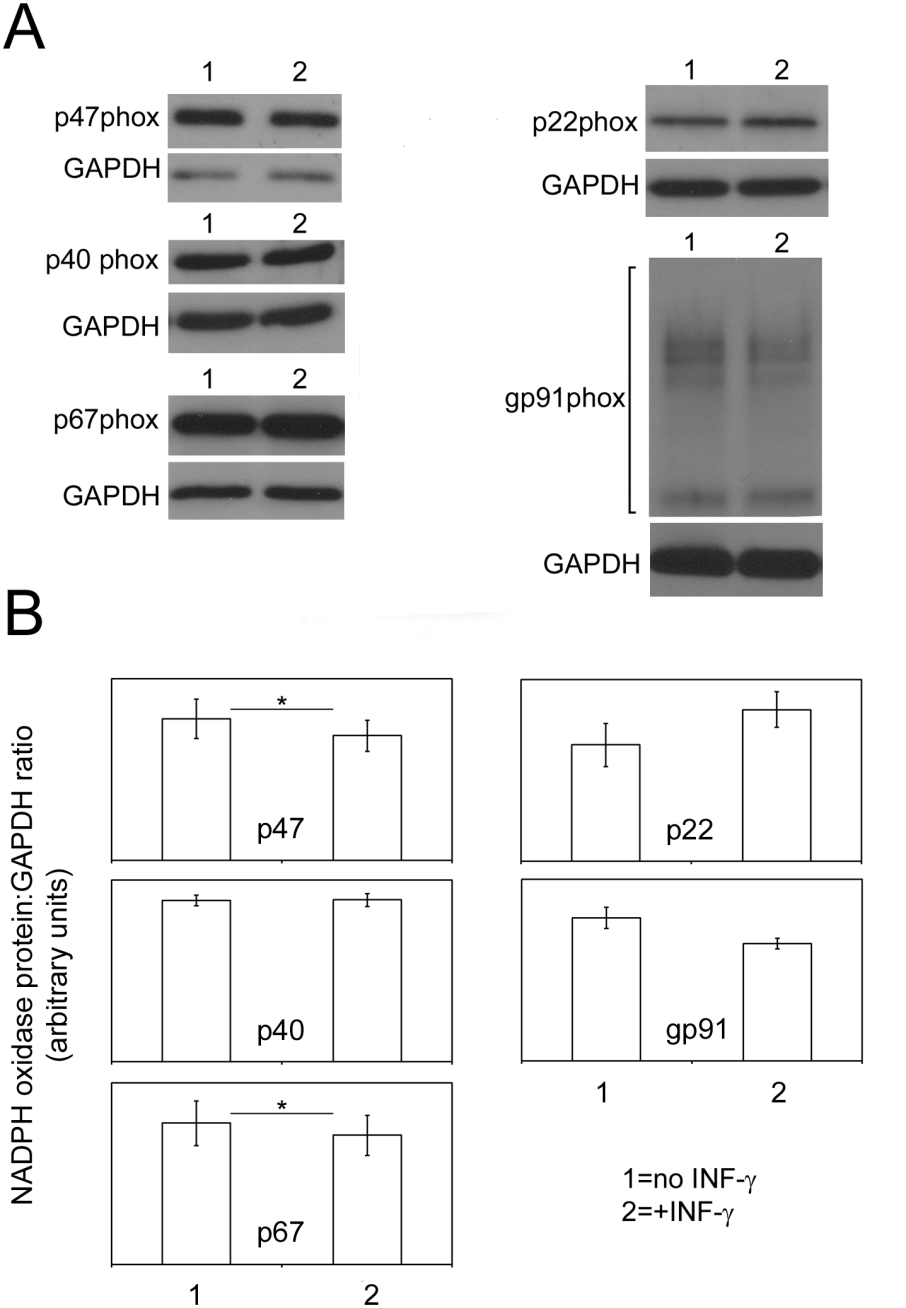
Changes in PLB-985 phox Proteins in Pre-Differentiated Cells Exposed to IFN-γ. A; The western blots are from representative experiments and show the indicated Nox2 proteins in PLB-985 cells that were pre-differentiated for 72 hours in media containing 1.3% DMSO and were then exposed for 3 hours to nothing (lane 1) or 30ng/ml IFN-γ (lane2). Western blotting for GAPDH was done to establish controls for gel loading and transfer efficiency. For gp91phox the protein was detected over a broad range of molecular weights (indicated by the square bracket) due to glycosylation. B; The graphs show the average density of phox proteins measured in 4 experiments like the representative examples in A; the protein densities are expressed as a ratio to the corresponding GAPDH density. For densitometry of gp91phox the lanes were scanned to the extent indicated by the square bracket in the representative gp91phox blot. The error bars are +/- SEM. * indicates a significant difference (p<0.05 by paired t-test).

To explore whether this smaller enhancement was due to changes in Nox2 proteins, pre-differentiated PLB-985 cells were treated with IFN-γ for 3 hours, RNA was extracted from these cells, and untreated controls, and was analyzed using microarrays. No significant changes in gp91phox, p22phox, p47phox, p67phox or p40phox mRNAs were seen when comparing the control and IFN-γ treated cells (ANOVA derived p values were greater than 0.05 in all cases, n = 4, data not shown). We also used western blotting to determine the levels of various Nox2 proteins in these samples. As can be seen in Fig 4, short term application of IFN-γ did not lead to significant increases in any of the Nox2 proteins and in fact caused small but statistically significant decreases in p47phox and p67phox which, given the lack of changes in corresponding mRNAs, may be due to changes in protein stability or reduced translation of the corresponding mRNAs.

## Discussion

Previously IFN-γ has been shown to modulate Nox2 activity and the expression of some of the phox proteins that make up Nox2; it has been reported that, under *in vitro* exposure of PMNs to IFN-γ, expression of gp91phox mRNA is increased while that of p47phox is diminished [17,18]. In addition, administration of IFN-γ to patients with CGD has been shown to decrease the incidence of severe infections [2] and IFN-γ is a key component in the management of patients with CGD [2,7,19]. In spite of this, little is known about the *in vivo* effect of IFN-γ on other Nox2 components, other non-Nox2 proteins that might alter PMN function or on PMN phenotypes such as chemotaxis, ingestion and microbial killing. Although IFN-γ has been proposed for use in other PMN dysfunction disorders, information is lacking about its efficacy in diseases other than CGD, osteopetrosis, hyperimmunoglobulin E syndromes and rare defects in the IL-12/IFN-γ pathways [1,2]. Furthermore, most studies evaluating effects of IFN-γ document changes for mature PMN incubated with IFN-γ rather than studying cells which have been differentiated under the influence of this cytokine [15,16,20,21].

In this study we have shown that IFN-γ has a dramatic effect on Nox2 activity when present during the differentiation of PLB-985 cells to a mature-PMN-like state (Fig 1). We found that Nox2 activity was not present in PLB-985 cells that had been exposed to IFN-γ for 72 or 96 hours and that DMSO treatment for the same times caused a wellknown induction of Nox2 activity. When IFN-γ was included with DMSO a further increase in O_2_^-^ production was observed; Nox2 activation by fMLF or PMA resulted in O_2_^-^ production that was approximately tripled in DMSO plus IFN-γ treated cells relative to DMSO treated cells.

In contrast to our finding that Nox2 activity was not induced in PLB-985 cells that had been exposed to IFN-γ for 72 or 96 hours, previous work [8,9] indicates that IFN-γ alone, at concentrations similar to those used in this study, induces Nox2 activity after only 2 days of application to the myeloid HL-60 cell line. Cell type differences between HL-60 and PLB-985 cells might account for the different findings of the earlier work and our current study.

In spite of being described as isolated years apart from different patients, DNA fingerprinting has shown that HL-60 [5] and PLB-985 [3] cells are from the same individual [6], indicating that the original “isolation” of PLB-985 cells probably reflects expansion of contaminating HL-60 cells. Thus PLB-985 cells are a subclone of the HL-60 line. Nevertheless, according to the original descriptions of PLB-985 and HL-60 cells, the two lines have very different granule morphology and distinct susceptibilities to cytological stains which suggests that PLB-985 cells are less matured along the myeloid lineage than HL-60 cells [3]. This phenotype may have been acquired over time in lab culture but is consistent with PLB-985 cells being a model for a more primitive precursor along the myeloid lineage. As outlined in Methods we confirmed phenotypic differences between the two lines consistent with a less matured phenotype for PLB-985 cells. Our current study indicates PLB-985 cells do not develop a functional Nox2 in response to IFN-γwhereas others [8,9] have shown that HL-60 cells do (which we have confirmed in our hands, see Methods). The divergent results may thus reflect the different effects of IFN-γ when applied to the less matured myeloid precursors modeled by PLB-985 cells.

In an attempt to model earlier work involving brief IFN-γ application to mature PMNs, we applied different IFN-γ concentrations, for 3 hours, to pre-differentiated PLB-985 cells and found that this was far less effective at enhancing O_2_^-^ production than including IFN-γ during the differentiation period (Fig 2). The application of IFN-γ to already differentiated cells failed to significantly enhance fMLF induced Nox2 activity but it did boost PMA mediated O_2_^-^ production; maximal enhancement was at concentrations of more than 15ng/ml and at these concentrations it increased O_2_^-^ production by about 10 nmol over the course of the experiment. Very different results were obtained when IFN-γ was co-applied for 72 hours during DMSO mediated differentiation. Co-application of IFN-γ and DMSO enhanced both fMLF and PMA mediated O_2_^-^ production maximally at concentrations of more than 15ng/ml. IFN-γ increased fMLF mediated O_2_^-^ production by 8 nmol over the course of the experiment and it boosted PMA mediated O_2_^-^ production by close to 3 fold more than what was seen with application to pre-differentiated cells. This data indicates that IFN-γ has a very different effect on the Nox2 enzyme of PLB-985 cells when applied during cell maturation versus being briefly applied to already mature cells.

To explore whether IFN-γ applied during PLB-985 cell differentiation enhances Nox2 activity because of increased expression of protein subunits of the Nox2 enzyme, we treated PLB-985 cells with IFN-γ, DMSO, or both, for 72 hours and carried out western blots to detect the Nox2 components p40phox, p47phox, p67phox, p22phox and gp91phox (Fig 3); whole-cell lysates and subcellular plasma membrane preparations were examined. A detailed description of the findings is outlined under Results and taken together they support the following explanations for the observed Nox2 activities.

The western blots indicate that whole-cell lysates from PLB-985 cells treated with IFN-γ have large increases in p22phox and gp91phox and smaller increases in p40phox, p47phox and p67phox relative to untreated cells. However, elevated p22phox and gp91phox were not seen in membrane preparations indicating a failure of cytochrome b558 heterodimer to properly assemble in membrane in this condition. This, and the very low level of the essential Nox2 subunit p47phox, even after the IFN-γ mediated increase, likely explains the inability of the IFN-γ alone to drive development of Nox2 activity.

In the presence of DMSO, whole-cell lysate levels of the two membrane associated Nox2 proteins were unchanged (for gp91phox) or were slightly reduced (for p22phox) relative to no treatment and appeared unchanged in plasma membrane. However p40phox, p47phox and p67phox levels were all increased dramatically relative to no treatment. Because these proteins are important for Nox2 function, the increase in their levels, particularly the large increase in p47phox, likely explains the fact that DMSO differentiated PLB-985 cells have active Nox2.

Inclusion of IFN-γ with DMSO was found to increase whole-cell lysate levels of p47phox, p22phox and gp91phox above the levels seen when only DMSO was present. Furthermore, the plasma membrane levels of p22phox and gp91phox showed corresponding large increases. Thus, elevation of the two membrane associated Nox2 subunits and p47phox are likely to contribute to the ability of IFN-γ to enhance Nox2 activity above the level induced by DMSO alone.

To determine whether the changes in Nox2 proteins that we observed were due to changes in transcription of the corresponding genes, we inferred their mRNA levels from DNA microarray data obtained when total RNA from PLB-985 cells, treated for 72 hours with IFN-γ, DMSO, or both, was applied to Affymetrix Human Genome 2.0 DNA microarrays (Table 1). Treatment with IFN-γ resulted in a significant 2.48 fold increase in gp91phox mRNA which is consistent with the large increase in gp91phox protein that we observed. In contrast, no increase in p22phox mRNA was seen suggesting that the large increase in p22phox protein upon IFN-γ treatment is due to stabilization of p22phox protein or elevated translation of gp22phox mRNA. Of these two possibilities we suggest that the former is likely correct given that many studies [22–27] show that gp91phox and p22phox mutually stabilize each other; in particular 1) the proteins are tightly associated, 2) in X-linked CGD, mutation of the gp91phox gene results in loss of gp91phox and also p22phox and, 3) in autosomal recessive CGD, mutation of the p22phox gene results in loss of p22phox and also gp91phox.

Treatment with IFN-γ alone also significantly increased (1.68 fold) p67phox mRNA which is consistent with the observed increase in p67phox protein. In contrast, although p40phox protein was increased by roughly the same amount as p67phox after IFN-γ treatment, its mRNA levels did not change by a statistically significant amount. The large increase in p40phox protein upon IFN-γ treatment may be because of increased protein stability or elevated translation of p40phox mRNA. We favor the former possibility given that p40phox is associated with p67phox and it is present in reduced amounts in patients with CGD lacking p67phox [28,29]. We propose that the increased levels of p67phox protein mediated by IFN-γ leads to an increased fraction of p40phox being bound to p67phox and thus p40phox stabilization.

Similarly, in IFN-γ treated cells relative to untreated cells, the small but statistically significant increase in p47phox protein (Fig 3) that occurred in the absence of an increase in p47phox mRNA (Table 1) may be explained by stabilization of p47phox protein as a result of its increased interaction with the more abundant p67phox protein.

Treatment of PLB-985 cells with DMSO caused large increases in the levels of all phox protein mRNAs. For the cytoplasmic proteins this correlates well with the large increases in their protein levels in whole-cell lysates. However, despite the large increases in their mRNAs, in whole-cell lysates gp91phox protein was unchanged and p22phox protein was down slightly relative to no treatment. Additionally, both proteins appeared unchanged in membrane fractions relative to no treatment. The failure of increased gp91phox and p22phox transcription to cause correspondingly large increases in their proteins indicates that a post translational effect of DMSO, such as increased protein instability, might limit the level of the cytochrome b558 complex during DMSO mediated differentiation. Reduced translation of the mRNAs of the membrane bound phox proteins could also explain this observation.

PLB-985 cells treated with DMSO plus IFN-γ displayed large increases in the levels of all phox protein mRNAs relative to untreated cells. However, for gp91phox and p47phox the increases in mRNA levels were larger than the increases with DMSO alone, gp91phox mRNA was elevated about 50% relative to DMSO alone and gp47phox mRNA was up almost 20%. These increases likely account for the elevation of gp91phox (in both whole cell lysates and membrane) and p47phox protein, relative to treatment with DMSO alone, when IFN-γ is included during cell differentiation. In contrast, treatment with DMSO plus IFN-γdid not increase p22phox mRNA relative to treatment with DMSO alone, despite the corresponding increases in p22phox protein. As discussed above, we favor the hypothesis that the increase in p22phox protein observed in this situation (Fig 3) is due to its stabilization by increased hetero-dimerization with the elevated amounts of gp91phox protein.

In conclusion, the increases in gp91phox, p22phox and p47phox protein likely account for at least some of the increase in Nox2 activity when PLB-985 cells are differentiated in the presence of IFN-γ and our microarray data indicates that these protein increases are likely mediated by increased transcription of the gp91phox and p47phox genes and a consequent stabilization of p22phox.

A noteworthy feature of the data is that the effects of IFN-γ on cytoplasmic phox protein expression are dependent on whether the PLB-985 cells are undergoing differentiation or not. A 72 hour addition of IFN-γ increases p67phox and p40phox protein (Fig 3) and increases p67phox transcription (Table 1) but has a very small effect on p47phox protein. In contrast, a 72 hour application of IFN-γ in the presence of DMSO increases p47phox transcription and protein relative to DMSO alone, but has no effect on p67phox or p40phox transcription or protein beyond what is seen with DMSO alone. Thus, changes induced by DMSO differentiation modulate changes induced by IFN-γ suggesting interplay between cell differentiation and the response to the cytokine.

We cannot rule out the possibility that increased Nox2 activity induced by IFN-γ is partially due to factors other than up regulation of Nox2 subunits. Some of the enhancement in fMLF stimulated Nox2 activity induced by IFN-γ may be due to increased expression of fMLP receptors or molecules that are involved in signaling from these receptors to the Nox2. Enhancement of the PMA response might be partially due to elevation of protein kinase C enzymes, G-protein coupled receptors, lipases and other enzymes which might be activated by PMA and which are stimulatory for Nox2 [30]. Such possibilities await genome wide transcription and proteomic analysis.

When IFN-γ was applied to pre-differentiated PLB-985 cells for 3 hours, a very different pattern of Nox2 protein expression was seen as compared to when IFN-γ was applied during differentiation (Fig 4). Slight *reductions* in p47phox and p67phox protein were observed and the other phox proteins were unchanged. Furthermore, microarray analysis indicated no significant changes in gp91phox, p22phox, p47phox, p67phox or p40phox mRNAs when comparing the control and IFN-γ treated cells (ANOVA derived p values were greater than 0.05 in all cases). Thus the slight reductions in p47phox and p67phox protein may be due to changes in protein stability or reduced translation of the corresponding mRNAs. The observation that an increase in PMA mediated O_2_^-^ production was seen in this experiment (Fig 2), albeit a much reduced increase relative to the one obtained by co-application of IFN-γ during maturation, might be due to increased expression of PMA activated, Nox2 stimulating molecules, as noted above [30].

The results of this study suggest that the influence of IFN-γ on myeloid cells as they differentiate to a mature state may be very different from its *in vitro* effects on already mature cells [15,16,20,21]. This work also indicates that IFN-γ has strong effects on phox protein expression and that these effects are different in cells undergoing terminal differentiation. Understanding the changes induced by IFN-γ during development of PMNs and other phagocytes may expand therapeutic strategies and uses for this cytokine in a variety of human disorders.
